# Supplementary material for: Impact of Protein-Enriched Plant Food Items on the Bioaccessibility and Cellular Uptake of Carotenoids
Source: Antioxidants (Basel). 2021 Jun 23;10(7):1005. doi: 10.3390/antiox10071005 (PMC8300660; doi:10.3390/antiox10071005)
Supplement: Supplementary file 1 [file antioxidants-10-01005-s001.zip › antioxidants-1266211-supplementary.pdf]

Table S1. The bioaccessibility of total carotenoids[1] from plant food matrices in the absence or presence of various proteins following *in vitro* gastrointestinal digestion. Tomato juice, spinach, and carrot juice were digested in the absence or presence of different protein-rich food items or supplements (Cod, turkey, WPI, SPI, SC, and GEL) at a concentration of 50% of protein recommended dietary allowance (RDA[2]). Values represent means ± SD of $n \geq 4$ replicates and $N \geq 2$ (sets repeated at different days). Values in a row represented without a common alphabetic letters differ significantly, $P < 0.05$.

	None (%)	Cod (%)	Turkey (%)	WPI (%)	SPI (%)	SC (%)	GEL (%)	P value[3]
**Spinach**	19,0 ±0.4^{A}	16.6 ±0.0^{B}	20.6 ±0.9^{C}	16.9 ±0.3^{B}	14.1 ±0.1^{D}	15.5 ±0.4^{B}	13.9 ±0.3^{D}	P<0.001
**Tomato juice**	2.3 ±0.3^{A}	2.3 ±0.3	1.6 ±0.0^{C}	2.9 ±0.1^{D}	3.3 ±0.0^{D}	3.0 ±0.0^{D}	3.0 ±0.1^{D}	P<0.001
**Carrot juice**	12.1 ±0.3^{A,B}	9.6 ±0.4^{C}	13.0 ±0.8^{B}	4.4 ±0.1^{D}	7.6 ±0.1^{E}	10.3 ±0.3^{C}	11.1 ±0.1^{A,C}	P<0.001

[1]Bioaccessibility is expressed as the percentage of total carotenoids recovered from the aqueous micellar fraction at the end of the *in vitro* GI digestion, compared to the amount present in each matrix at the beginning of digestion.

[2]RDA: corresponding to approx. 60 g/d for human adults [34].

[3]Fisher F-test followed by Bonferroni post-hoc tests.

Table S2. Average bioaccessibility[1] (± SD) of carotenoids from plant food matrices co-digested with different proteins at varying concentrations[2]. Values represent means of n≥4 replicates and N≥2 (sets repeated at different days). Values labelled with either *(P < 0.05) or **(P < 0.001) were significantly different from the control condition (no added protein).

			Carotenoid bioaccessibility (%)								
**Matrix**	**Prot. type**	**Prot. conc. %**[3]	**Neo**	**Vio**	**Zea+Lut**	**PTF**	**PTE**	**α-Car**	**β-Car**	**Lyc**	**Tot. car.**
**Spinach**	None	0	22,1 ±0.5	2,8 ±0.1	37,5 ±0.9	n.d.	n.d.	n.d.	3,3 ±0.1	n.d.	19,0 ±0.4
	WPI	10	18,8 ±0.4**	2.4 ±0.1*	37.1 ±1.2	n.d.	n.d.	n.d.	3.9 ±0.1**	n.d.	18.5 ±0.5
		25	18,0 ±0.4**	2,5 ±0.0*	35.4 ±0.6	n.d.	n.d.	n.d.	4.6 ±0.0**	n.d.	17,9 ±0.3
		50	15,1 ±0.4**	2,2 ±0.1**	32.8 ±0.7*	n.d.	n.d.	n.d.	6.7 ±0.1**	n.d.	16.9 ±0.3*
	SPI	10	24,0 ±0.5	3,2 ±0.1*	36,7 ±0.9	n.d.	n.d.	n.d.	5.2 ±0.1**	n.d.	19,5 ±0.5
		25	17,2 ±0.2**	2,4 ±0.0*	29,6 ±1.0**	n.d.	n.d.	n.d.	7,5 ±0.1**	n.d.	16,2 ±0.4**
		50	11,6 ±0.1**	1.8 ±0.1**	23.7 ±0.1**	n.d.	n.d.	n.d.	12,2 ±0.2**	n.d.	14.1 ±0.1**
	SC	10	23,5 ±0.5	3,0 ±0.1	33,1 ±0.2*	n.d.	n.d.	n.d.	2.9 ±0.1*	n.d.	17,5 ±0.1
		25	22,2 ±0.1	2,6 ±0.0	32,9 ±0.4*	n.d.	n.d.	n.d.	2,5 ±0.1**	n.d.	17,0 ±0.2*
		50	18,6 ±0.3**	2.6 ±0.1	30,5 ±0.9**	n.d.	n.d.	n.d.	2,5 ±0.1**	n.d.	15.5 ±0.4**
	GEL	10	22,1 ±0.6	2,8 ±0.1	37,1 ±0.9	n.d.	n.d.	n.d.	3.3 ±0.1	n.d.	18,9 ±0.4
		25	16,3 ±0.3**	2.7 ±0.0	33,2 ±0.8*	n.d.	n.d.	n.d.	2.8 ±0.0*	n.d.	16,4 ±0.3*
		50	13.2 ±0.2**	3,0 ±0.1	28,8 ±0.8**	n.d.	n.d.	n.d.	1.4 ±0.1**	n.d.	13.9 ±0.3**
**Tomato juice**	None	0	n.d.	n.d.	66,5 ±1.4	5,1 ±0.1	7,6 ±0.1	n.d.	8,0 ±0.2	0,3 ±0.0	2.3 ±0.3
	WPI	10	n.d.	n.d.	65.9 ±2.1	8,6 ±0.2**	15,3 ±0.3**	n.d.	11,6 ±0.3**	0.5 ±0.0**	4.1 ±0.1**
		25	n.d.	n.d.	65,4 ±1.8	10,6 ±0.4**	18,6 ±0.4**	n.d.	14.5 ±0.2**	0.4 ±0.0**	4.8 ±0.1**
		50	n.d.	n.d.	61,3 ±1.1	6,1 ±0.2*	11,9 ±0.4**	n.d.	11.5 ±0.1**	n.d.	2.9 ±0.1**
	SPI	10	n.d.	n.d.	59.8 ±0.4*	11.0 ±0.3**	14.4 ±0.2**	n.d.	15.5 ±0.5**	0.6 ±0.0**	4.3 ±0.1**
		25	n.d.	n.d.	44.5 ±0.8**	11.9 ±0.1**	13.1 ±0.3**	n.d.	16.0 ±0.3**	0.5 ±0.0**	4.1 ±0.0**
		50	n.d.	n.d.	29.8 ±0.8**	8.8 ±0.1**	11.1 ±0.3**	n.d.	13.7 ±0.4**	0.4 ±0.0**	3.3 ±0.0**
	SC	10	n.d.	n.d.	65.9 ±0.6	5.0 ±0.1	7.4 ±0.1	n.d.	7.8 ±0.0	0.3 ±0.0	2.3 ±0.0
		25	n.d.	n.d.	71.4	11,0	12.9	n.d.	19,7	0.5	4.2

					±0.7	±0.3**	±0.3**		±0.4**	±0.0**	±0.1**
		50	n.d.	n.d.	76,0 ±2.3*	6.9 ±0.2**	8,4 ±0.2*	n.d.	17,0 ±0.4**	0.4 ±0.0**	3.0 ±0.0**
	GEL	10	n.d.	n.d.	61.9 ±1.5	4.7 ±0.1	7.9 ±0.2	n.d.	7.4 ±0.2	0.2 ±0.0*	2.2 ±0.1
		25	n.d.	n.d.	68.2 ±1.1	6.0 ±0.2*	9.7 ±0.2*	n.d.	9.8 ±0.3**	0.3 0.0	2,8 ±0.0*
		50	n.d.	n.d.	63.1 ±1.2	6.2 ±0.1**	10.3 ±1.6**	n.d.	12,7 ±0.1**	0.3 ±0.0	3.0 ±0.1**
**Carrot juice**	None	0	n.d.	n.d.	61,9 ±1.2	35,6 ±1.6	53,4 ±1.4	4,8 ±0.1	4,7 ±0.2	n.d.	12.1 ±0.3
	WPI	10	n.d.	n.d.	56,3 ±1.5*	19,4 ±0.6**	31,5 ±0.8**	1,8 ±0.1**	1,4 ±0.0**	n.d.	6,1 ±0.1**
		25	n.d.	n.d.	55,2 ±0.8*	16,2 ±0.4**	26,4 ±0.7**	1,5 ±0.0**	1,0 ±0.0**	n.d.	5,1 ±0.1**
		50	n.d.	n.d.	54,2 ±0.7*	12,1 ±0.2**	23,8 ±1.6**	1,2 ±0.0**	1,0 ±0.0**	n.d.	4.4 ±0.1**
	SPI	10	n.d.	n.d.	51,7 ±1.6*	18,8 ±0.4**	37,7 ±1.1**	2,6 ±0.1**	3,4 ±0.1**	n.d.	7,8 ±0.2**
		25	n.d.	n.d.	44,9 ±0.3**	18,7 ±0.3**	39,0 ±0.4**	2,6 ±0.1**	3,4 ±0.1**	n.d.	7,7 ±0.1**
		50	n.d.	n.d.	34,4 ±1.0**	18,3 ±0.1**	38,4 ±0.9**	2,6 ±0.0**	3,5 ±0.1**	n.d.	7.6 ±0.1**
	SC	10	n.d.	n.d.	64,3 ±1.1	45,3 ±1.1**	69,8 ±1.8**	6,1 ±0.0**	6,3 ±0.0**	n.d.	15,6 ±0.2**
		25	n.d.	n.d.	63,1 ±0.4	40,6 ±0.9*	68,6 ±2.1**	5,9 ±0.1**	6,2 ±0.1**	n.d.	14,8 ±0.2**
		50	n.d.	n.d.	60,1 ±0.4	29,6 ±1.0**	51,6 ±1.8	3,1 ±0.1**	3,7 ±0.1**	n.d.	10.3 ±0.3**
	GEL	10	n.d.	n.d.	59,9 ±0.6	27,4 ±0.8**	49,4 ±0.7	4,1 ±0.3*	3,9 ±0.1**	n.d.	10,2 ±0.2**
		25	n.d.	n.d.	57,5 ±0.6*	29,6 ±0.8**	48,9 ±1.3	4,2 ±0.3*	3,6 ±0.1**	n.d.	10,3 ±0.1**
		50	n.d.	n.d.	51,3 ±0.6**	34,2 ±0.5	51,0 ±0.4	4,1 ±0.1*	4,0 ±0.1*	n.d.	11.1 ±0.1*

[1]Bioaccessibility expressed as the percentage of carotenoids recovered from the aqueous micellar fraction at the end of the *in vitro* GI digestion, compared to the initial amount present in the undigested test meal.
[2]Concentrations given in final digestion volume are 0, 10, 25, and 50% based-RDA (approx. 60 g/d for human adults [34]).
[3]RDA: recommended dietary allowance; WPI: whey protein isolate; SPI: soy protein isolate; SC: sodium caseinate; GEL: gelatin; Neo: neoxanthin; Vio: violaxanthin; Zea+Lut: lutein+zeaxanthin; PTF: phytofluene; PTE: phytoene; α-Car: α-carotene; β-Car: β-carotene; Lyc: lycopene. n.d.: not detectable.

Table S3. Average cellular uptake[1] (%, ±SD) of carotenoids from plant food matrices co-digested with different proteins at varying concentrations[2]. Values represent means of n=3 replicates. Values labelled with *($P < 0.05$) were significantly different from the control condition (no added protein).

Matrix	Prot. type	Prot. conc. %[3]	Neo+Vio	Zea+Lut	β-Cry	PTF	PTE	α-Car	β-Car	Lyc	Tot. car.
Spinach	None	0	0.5 ±0.03	2.5 ±0.17	0.05 ±0.0	n.d.	n.d.	0.02 ±0.0	0.5 ±0.04	n.d.	1,3 ±0,08
	WPI	10	0.4 ±0.0	2.6 ±0.05	0.05 ±0.0	n.d.	n.d.	0.02 ±0.0	0.5 ±0.01	n.d.	1,3 ±0,02
		25	0.5 ±0.0	3.0 ±0.11	0.06 ±0.0	n.d.	n.d.	0.03 ±0.0	0.7 ±0.01	n.d.	1,5 ±0,04
		50	0.3 ±0.0*	2.5 ±0.03	0.05 ±0.0	n.d.	n.d.	0.02 ±0.0	0.5 ±0.0	n.d.	1,2 ±0,01
	SPI	10	0.4 ±0.0	2.3 ±0.06	0.05 ±0.0	n.d.	n.d.	0.02 ±0.0	0.7 ±0.02*	n.d.	1,2 ±0,03
		25	0.3 ±0.0**	2.3 ±0.09	0.05 ±0.0	n.d.	n.d.	0.03 ±0.0	0.8 ±0.02*	n.d.	1,2 ±0,04
		50	0.1 ±0.0**	1.2 ±0.08**	0.04 ±0.0	n.d.	n.d.	0.02 ±0.0	0.4 ±0.02*	n.d.	0,6 ±0,04**
	SC	10	0.5 ±0.03	2.4 ±0.21	0.05 ±0.0	n.d.	n.d.	0.02 ±0.0	0.5 ±0.03	n.d.	1,2 ±0,1
		25	0.3 ±0.01*	2.3 ±0.03	0.06 ±0.0	n.d.	n.d.	0.03 ±0.0	0.7 ±0.02	n.d.	1,2 ±0,02
		50	0.3 ±0.01*	2.3 ±0.13	0.06 ±0.0	n.d.	n.d.	0.03 ±0.0	0.7 ±0.04	n.d.	1,2 ±0,06
	GEL	10	0.7 ±0.03*	3.5 ±0.15*	0.04 ±0.0	n.d.	n.d.	0.03 ±0.0	0.9 ±0.04*	n.d.	1,8 ±0,07*
		25	0.4 ±0.01	3.2 ±0.19	0.03 ±0.0*	n.d.	n.d.	0.02 ±0.0	0.4 ±0.02	n.d.	1,5 ±0,07
		50	0.3 ±0.02**	3.2 ±0.27	0.03 ±0.0*	n.d.	n.d.	0.02 ±0.0	0.6 ±0.04	n.d.	1,5 ±0,12
Tomato juice	None	0	n.d.	17.1 ±0.83	n.d.	1.2 ±0.06	1.4 ±0.09	1.8 ±0.08	7.6 ±0.3	0.09 ±0.01	0,7 ±0,03
	WPI	10	n.d.	19.7 ±0.4	n.d.	1.5 ±0.05	1.7 ±0.09	2.3 ±0.05**	9.5 ±0.2*	0.3 ±0.0**	0,9 ±0,0*
		25	n.d.	28.1 ±0.7**	n.d.	2.1 ±0.07**	2.1 ±0.14	2.7 ±0.04**	12.7 ±0.4**	0.3 ±0.01**	1,1 ±0,04**
		50	n.d.	26.9 ±0.4**	n.d.	2.2 ±0.04**	3.3 ±0.11**	2.3 ±0.04**	21.1 ±0.1**	0.5 ±0.01**	1,5 ±0,04**
	SPI	10	n.d.	25.8 ±0.3**	n.d.	2.3 ±0.03**	2.6 ±0.08**	2.0 ±0.03	13.3 ±0.03**	0.16 ±0.01**	1,2 ±0,02**
		25	n.d.	32.8 ±0.8**	n.d.	2.8 ±0.09**	3.3 ±0.04**	2.6 ±0.04**	17.8 ±0.25**	0.12 ±0.0**	1,4 ±0,02**
		50	n.d.	27.4 ±0.9**	n.d.	4.1 ±0.14**	4.1 ±0.31**	3.2 ±0.1**	23.2 ±0.75**	0.33 ±0.01**	1,9 ±0,08**
	SC	10	n.d.	11.5 ±0.2*	n.d.	0.8 ±0.03*	1.0 ±0.07	1.6 ±0.07	5.7 ±0.2*	0.06 ±0.0*	0,5 ±0,02*
		25	n.d.	20.6 ±0.8	n.d.	1.7 ±0.13	1.5 ±0.18	1.6 ±0.08	7.9 ±0.2	0.08 ±0.0	0,7 ±0,05

		50	n.d.	9.1 ±0.4**	n.d.	1.0 ±0.06	1.0 ±0.1	1.6 ±0.08	5.0 ±0.13**	0.12 ±0.0	0,5 ±0,02
	GEL	10	n.d.	20.3 ±0.6*	n.d.	1.3 ±0.03	1.4 ±0.17	2.3 ±0.06**	9.3 ±0.26*	0.06 ±0.0**	0,7 ±0,03
		25	n.d.	17.9 ±0.7	n.d.	1.1 ±0.03	1.3 ±0.08	2.1 ±0.03*	8.5 ±0.25	0.08 ±0.0	0,6 ±0,02
		50	n.d.	23.9 ±0.3**	n.d.	2.7 ±0.09**	3.3 ±0.28**	3.1 ±0.05**	14.6 ±0.24**	0.15 ±0.0**	1,3 ±0,04**
**Carrot juice**	None	0	n.d.	3.6 ±0.14	n.d.	0.2 ±0.05	n.d.	0.05 ±0.0	0.13 ±0.0	0.05 ±0.01	0,2 ±0,0
	WPI	10	n.d.	4.3 ±0.13	n.d.	0.2 ±0.01	n.d.	0.05 ±0.0	0.13 ±0.0	0.03 ±0.0	0,2 ±0,0
		25	n.d.	3.7 ±0.07	n.d.	0.2 ±0.01	n.d.	0.08 ±0.0	0.17 ±0.0*	0.03 ±0.0	0,2 ±0,0
		50	n.d.	1.8 ±0.19**	n.d.	0.01 ±0.01**	n.d.	0.05 ±0.01	0.09 ±0.01**	0.16 ±0.13	0,09 ±0,01*
	SPI	10	n.d.	3.9 ±0.18	n.d.	0.3 ±0.02*	n.d.	0.08 ±0.01*	0.18 ±0.01*	0.06 ±0.01	0,2 ±0,01
		25	n.d.	2.5 ±0.04*	n.d.	0.2 ±0.01	n.d.	0.05 ±0.0	0.11 ±0.0	0.06 ±0.01	0,12 ±0,0
		50	n.d.	3.0 ±0.2	n.d.	0.1 ±0.01	n.d.	0.05 ±0.0	0.11 ±0.01	0.07 ±0.01	0,13 ±0,01
	SC	10	n.d.	8.6 ±0.4**	n.d.	0.7 ±0.04**	n.d.	0.11 ±0.01**	0.28 ±0.0**	0.09 ±0.01	0,4 ±0,02**
		25	n.d.	4.5 ±0.07*	n.d.	0.3 ±0.0**	n.d.	0.04 ±0.0*	0.13 ±0.0	0.07 ±0.01	0,2 ±0,0
		50	n.d.	3.4 ±0.09	n.d.	0.2 ±0.01	n.d.	0.04 ±0.0*	0.10 ±0.0*	0.14 ±0.08	0,14 ±0,0
	GEL	10	n.d.	3.5 ±0.07	n.d.	0.2 ±0.01	n.d.	0.06 ±0.0	0.12 ±0.0	0.05 ±0.01	0,15 ±0,0
		25	n.d.	3.3 ±0.02	n.d.	0.2 ±0.02	n.d.	0.06 ±0.0	0.15 ±0.0*	0.05 ±0.0	0,16 ±0,0
		50	n.d.	2.9 ±0.02**	n.d.	0.2 ±0.01	n.d.	0.10 ±0.0**	0.19 ±0.0**	0.05 ±0.0	0,20 ±0,0**

[1]Cellular uptake represents the fraction of carotenoid recovered in the Caco-2 cell fraction compared to the original matrix content, considering all dilutions.
[2]Concentrations given in final digestion volume are 0, 10, 25, and 50% based-RDA (approx. 60 g/d for human adults [34]).
[3]RDA: recommended dietary allowance; WPI: whey protein isolate; SPI: soy protein isolate; SC: sodium caseinate; GEL: gelatin; Neo: neoxanthin; Vio: violaxanthin; Zea+Lut: lutein+zeaxanthin; PTF: phytofluene; PTE: phytoene; α-Car: α-carotene; β-Car: β-carotene; Lyc: lycopene. n.d.: not detectable.

Table S4. The MRM transitions scanned for detection of compounds of interest.

Compound	Retention time (min)	Parent ion	Quandtification transition (Da)	CE (V)	Confirmation transition (Da)	CE (V)	Confirmation transition (Da)	CE (V)	Confirmation transition (Da)	CE (V)
**Vio**	2.26	$[M+H]^+$	601.6 > 221.1	19	601.6 > 583.2	9	601.6 > 491.3	17		
**Neo**	2.31	$[M+H]^+$	601.6 > 167.2	15	601.6 > 583.2	9	601.6 > 221.1	19		
**Zea**	3.88	$[M]^{+\bullet}$	568.5 > 476.2	12	568.5 > 550.4	11	568.5 > 338.4	15		
**Lut**	3.91	$[M]^{+\bullet}$	568.5 > 338.4	15	568.5> 476.2	12	568.5 > 430.3	15		
**β-apo-Car**	4.50	$[M]^{+\bullet}$	416.4 > 324.3							
**β-Cry**	4.90	$[M]^{+\bullet}$	552.4 > 460.2	13	552.4 > 537.1	17				
**Lyc**	5.41	$[M]^{+\bullet}$	536.5 > 444.2	9	536.5 > 467.3	11	536.5 > 375	13		
**α-Car**	6.22	$[M]^{+\bullet}$	536.5 > 444.2	13	536.5 > 321.1	15	536.5 > 413.3	13		
**β-Car**	6.34	$[M]^{+\bullet}$	536.5 > 444.2	13	536.5 > 346.3	13	536.5 > 399.3	14	536.5 > 281	18
**(9Z)-β-Car**	6.38	$[M]^{+\bullet}$	536.5 > 444.2	13	536.5 > 346.3	13	536.5 > 399.3	14	536.5 > 281	18
**PTF**	6.43	$[M]^{+\bullet}$	542.5 > 337.2	13	542.5 > 404.9	11				
**PTE**	6.54	$[M]^{+\bullet}$	544.5 > 81	35	544.5 > 450.3	8	544.5 > 339.2	12		
